# Bioaccumulative characteristics of tetrabromobisphenol A and hexabromocyclododecanes in multi-tissues of prey and predator fish from an e-waste site, South China

**DOI:** 10.1007/s11356-015-4463-1

**Published:** 2015-04-15

**Authors:** Bin Tang, Yan-Hong Zeng, Xiao-Jun Luo, Xiao-Bo Zheng, Bi-Xian Mai

**Affiliations:** State Key Laboratory of Organic Geochemistry and Guangdong Key Laboratory of Environmental Resources Utilization and Protection, Guangzhou Institute of Geochemistry, Chinese Academy of Sciences, Guangzhou, 510640 People’s Republic of China; University of Chinese Academy of Sciences, Beijing, 100049 People’s Republic of China

**Keywords:** TBBPA, HBCDs, Tissue accumulation, Species-specific bioaccumulation, Diastereoisomer, Enantiomer

## Abstract

Tetrabromobisphenol A (TBBPA) and hexabromocyclododecanes (HBCDs) were analyzed in 12 tissues of prey (mud carp) and predator (northern snakehead) fish from an e-waste area, South China. The TBBPA concentrations in different tissues ranged from 0.03 to 2.85 ng/g wet weight (ww) in mud carp and 0.04 to 1.30 ng/g ww in northern snakehead. The concentrations of HBCDs ranged from 0.07 to 96.9 ng/g ww in mud carp and 0.18 to 240 ng/g ww in northern snakehead. HBCD levels in tissues were correlated with lipid content for both fish species, while this correlation was only found in mud carp for TBBPA. Meanwhile, northern snakehead exhibited higher HBCD levels but lower TBBPA levels than mud carps. These observations are attributed to the more polar and reactive properties of TBBPA than HBCDs. α-HBCD was the predominant diastereoisomer of HBCDs in all tissues of mud carp and northern snakehead, except for chyme of mud carp. All the analyzed tissues in mud carp showed an enrichment of (+)-α-HBCD enantiomer with EF (enantiomeric fraction) values of 0.53–0.62, but that in northern snakehead showed an enrichment of (−)-α-HBCD enantiomer with EF values of 0.35–0.5. Considering the fact that the mud carp is one of the diet items of northern snakehead, the different enantiomer accumulation characteristics of α-HBCD between the two fish species in the present study indicated that prey and predator fish could prefer to biotransform different enantiomers of α-HBCD.

## Introduction

Tetrabromobisphenol A (TBBPA) and hexabromocyclododecanes (HBCDs) are two classes of brominated flame retardants (BFRs) which had been used in a variety of commercial and industrial applications to enhance fire resistance (Morose 2006). TBBPA is most commonly used as a reactive flame retardant in epoxy resins for printed wiring boards, but it also has additive applications in several types of polymers (Birnbaum and Staskal 2004). HBCDs are used as an additive flame retardant incorporated in expanded polystyrene and extruded polystyrene application in building and construction industry (Covaci et al. 2003). Technical HBCDs are dominated by three diastereoisomers, α-, β-, and γ-HBCD with percentages of 10–13, 1–12, and 75–89 %, respectively (Law et al. 2005). Each of these diastereoisomers has two enantiomers.

TBBPA and HBCDs had been found to be ubiquitous in biota, from zooplankton to polar bears and humans (Johnson-Restrepo et al. 2008; Meng et al. 2012; Tomy et al. 2008), as well as in abiotic samples, such as water and sediments (Feng et al. 2012; Yang et al. 2012). TBBPA had been reported to affect thyroid hormones and neurological function (Van der Ven et al. 2008), and HBCDs had been shown to disrupt functioning of the thyroid hormone system in wildlife and cause oxidative damage to lipids, proteins, and DNA in animals (Marvin et al. 2011). Moreover, HBCDs was listed as a persistent organic pollutant at the 6th meeting of the Stockholm Convention on Persistent Organic Pollutants (UNEP 2013). It makes the occurrence of these pollutants to be a globally concerned issue.

Nowadays, e-waste recycling area has become a hot spot of contamination of halogenated organic pollutants (HOPs). Longtang town of Guangdong province in South China is one of the largest e-waste recycling regions in China (Zhang et al. 2012). Numerous studies have reported elevated levels of HOPs in both aquatic and terrestrial organisms collected from Longtang town due to the primitive e-waste recycling activities (Zhang et al. 2010; Zheng et al. 2012). Fish are usually used for environmental monitoring due to the direct pathway of accumulating pollutants from water and diet (Lanfranchi et al. 2006). Generally, studies have reported that the fate of some HOPs in fish was tissue-specific and species-specific (Guo et al. 2008). However, prior to this study, data on the occurrence of TBBPA and HBCDs in fish species were mostly focused on the fish muscle (Meng et al. 2012; Morris et al. 2004), and the distribution of these compounds in other tissues, e.g., fish scale, skin, gill or liver, was scarcely taken into consideration. Moreover, studies have suggested that TBBPA could be degraded in an aqueous medium (Liu et al. 2006), and HBCDs could also be diastereoisomer- and enantiomer-specific in bioisomerization in fish (Du et al. 2012).

Hence, the primary aim of this study was to investigate the tissue-specific and species-specific bioaccumulation of TBBPA and HBCDs in fish species with different dietary habits from an e-waste region. The prey fish (mud carp, *Cirrhinus molitorella*) and the predator fish (northern snakehead, *Ophicephalus argus*) were collected from a natural pond contaminated by e-waste in South China. Concentrations of TBBPA and HBCDs in the different tissues of the studied fish species were investigated. Also, the diastereoisomer and enantiomer compositions of HBCDs were examined to explore the metabolic process for HBCDs in the two fish species.

## Materials and methods

### Sampling

Sampling was conducted in 2010. A total of ten fish samples were collected, including five mud carp (30 ± 1 cm in length) and five northern snakehead (23 ± 2 cm in length), from a pond located in Longtang town, Qingyuan County, South China. Twelve tissues (the scale, skin, gill, liver, kidney, heart, bladder, muscle, roe, fat, intestine, and chyme) were collected from each species. The scale, skin, gill, liver, kidney, heart, bladder, and muscle were carefully taken using steel tweezers, scissors, and a medical blade from each sample. About 5 g (wet weight; ww) of fish muscle was taken from the fish’s back. The skin without muscle and the scale were obtained from both sides of the fish’s back. The gills and livers were washed to clear blood. The intestine and chyme of individual fish were carefully separated, and the intestine was cleaned by ultrapure water. Perivisceral adipose tissue (fat) was obtained from the visceral surface from the mud carp and northern snakehead, but fat were only found in four mud carp and three northern snakehead. Additionally, roe were obtained in three mud carp and three northern snakehead. Due to the little mass of tissues, heart, liver, kidney, bladder, intestine, and chyme both in mud carp and in northern snakehead, all tissues were pooled into one sample separately, and the fat, roe, and gill tissues in the northern snakehead were pooled into one sample. Finally, 57 tissue samples were obtained, and they were preserved at a temperature of −20 °C before analysis.

### Sample preparation

The sample preparation method for the separation of TBBPA and HBCDs was generally similar to the analytical procedures described in previous studies (Köppen et al. 2010), with some modifications. Briefly, freeze-dried samples were ground into powder and spiked with surrogate standards (^13^C-labeled α-, β-, γ-HBCD and ^13^C-labeled TBBPA) prior to Soxhlet extraction with a mixture of acetone:hexane (1:1, *v/v*) for 48 h. The extract was concentrated and then the solvent exchanged to hexane (10 mL). An aliquot of organic extract was evaporated to determinate the lipid content. The remainder was treated with concentrated H_2_SO_4_ to remove the lipids and then purified on a silica gel column (i.d. = 1.0 cm) packed with 12 cm neutral silica (3 % deactivated, *w/w*) topped with a 2-cm layer of anhydrous Na_2_SO_4_. The column was eluted with 13 mL hexane followed by 5 mL dichloromethane:hexane (1:1, *v/v*) and 45 mL dichloromethane:hexane (1:1, *v/v*). The first and the second fractions were discarded, and the last fraction was collected. Finally, the extract was evaporated to near-dryness and redissolved in 200 μL methanol, and internal standards (*d*_18_-labeled α-, β-, γ-HBCD) were added before liquid chromatography/mass spectrometry (LC/MS) analysis.

### Instrumental analysis

The analysis of TBBPA and HBCDs was performed on an Agilent 1200 series liquid chromatography (LC) system coupled to an Agilent 6410 electrospray triple quadrupole mass spectrometer. A XDB-C_18_ column (50 mm × 4.6 mm i.d., 1.8 μm, Agilent, CA) was used for TBBPA and HBCD diastereoisomer separation. A Phenomenex Nucleosil β-PM chiral LC column (200 mm × 4.0 mm i.d., 5 μm, Macherey-Nagel, GmbH & Co., Germany) was used for the HBCD enantiomer separation. Details of the analytical methodology used for separation and quantification of TBBPA and HBCD analysis were published previously (Feng et al. 2012).

Chiral HBCD compositions were expressed as enantiomeric fraction (EF), which is determined from the ratio of (+) enantiomer area over the sum peak areas of the (+) and (−) enantiomers corrected by areas of the corresponding *d*_18_-labeled diastereoisomer standards (Marvin et al. 2007).

### Quality assurance and quality control (QA/QC)

Quality assurance was performed by the analyses of procedural blanks, spiked blanks, and spiked matrices. Procedural blanks were analyzed. The recoveries of TBBPA and HBCDs in spiked blanks ranged from 74 to 97 % and 93 to 108 % and those in spiked matrices ranged from 84 to 99 % and 94 to 117 %. The method detection limits (MDLs) were defined as a signal to noise (*S/N*) ratio of 10. The MDLs were in the range of 16–25 pg/g (ww) for TBBPA and 32–52 pg/g ww for HBCDs. No TBBPA and HBCDs were detected in procedural blanks. The average recoveries of all the spiked surrogate standards were ranged from 78 to 112 % for TBBPA and 83 to 122 % for HBCDs.

## Results and discussion

### TBBPA levels in fish tissues

TBBPA levels in mud carp (mean value of 837 pg/g, ww) were significantly higher than those in northern snakehead (mean value of 384 pg/g, ww) when whole body (not accounting for tissues) was considered. Northern snakehead occupied a higher trophic level than mud carp. The lower TBBPA concentrations in northern snakehead than those in mud carp were likely due to higher metabolism capacity of northern snakehead since TBBPA is a more polar and reactive compound than other BFRs (Johnson-Restrepo et al. 2008).

The concentration of TBBPA in fish in previous studies was reported on a lipid-weight basis. Thus, a comparison of TBBPA concentrations in this study with other studies was conducted on a lipid-weight basis. The lipid normalized concentration of TBBPA in the muscles of mud carp (4.3 ng/g, lw) and of northern snakehead (9.7 ng/g, lw) were one to two orders of magnitude greater than those detected in the edible parts of the fish (0.01 ng/g for barracuda, 0.03 ng/g for mullet, 0.04 ng/g for seerfish, and 0.11 ng/g for sardine) from three regions of Japan (Ashizuka et al. 2008) and were much higher than those in mysid shrimp (*Neomysis integer*) (0.8 and 0.9 ng/g lw) from two sites in the Scheldt estuary (Verslycke et al. 2005). TBBPA levels in the present study were comparable with those in the muscles of perch, pike, smelt, vendace, and trout from Norway (1.0 to 13.7 ng/g lw) (Schlabach et al. 2004), in the blubber of bottlenose dolphin (0.451 to 1.86 ng/g lw) from the USA, in the muscle of bull shark (5.17 to 13.2 ng/g lw) from the USA (Johnson-Restrepo et al. 2008), and in a variety of aquatic biota from the North Sea (2.5 to 14 ng/g lw) (Morris et al. 2004). The TBBPA concentrations in the present study were much lower than those (28.5 to 39.4 ng/g lw) in four fish species from Lake Chaohu, located adjacent to two most developed regions in China (Yang et al. 2012).

**Fig. 1:**
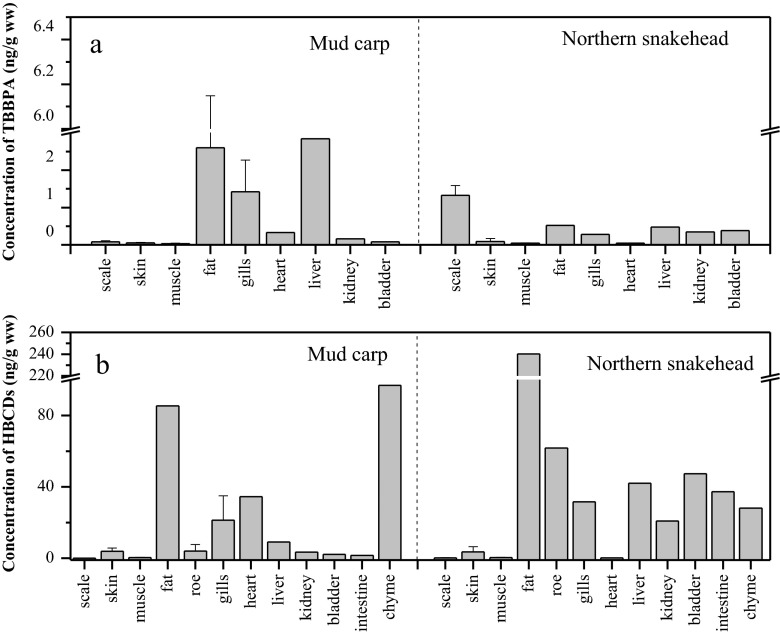
TBBPA (**a**) and ΣHBCD (**b**) concentrations in different tissues of mud carp and northern snakehead

The levels of TBBPA varied with tissues. No TBBPA were detected in the roe, intestine, and chyme in the two fish species. The concentrations of TBBPA in other tissues ranged from 0.03 to 2.85 ng/g (ww) for mud carp and 0.04 to 1.30 ng/g (ww) for northern snakehead (Table S1). TBBPA levels in mud carp follows the order of liver (2.85 ng/g, ww) ~ fat (2.60 ng/g, ww) > gill (1.42 ng/g, ww) > heart (0.33 ng/g, ww) > kidney (0.16 ng/g, ww) > scale (0.08 ng/g, ww), bladder (0.08 ng/g, ww), skin (0.05 ng/g, ww), and muscle (0.03 ng/g, ww) (Fig. 1a). For northern snakehead, the levels of TBBPA in tissues were as follows: scale (1.33 ng/g, ww) > fat (0.52 ng/g, ww) > liver (0.47 ng/g, ww) > bladder (0.38 ng/g, ww), kidney (0.34 ng/g, ww) and gills (0.28 ng/g, ww) > skin (0.08 ng/g, ww), muscle (0.04 ng/g, ww), and heart (0.04 ng/g, ww) (Fig. 1a).

**Fig. 2:**
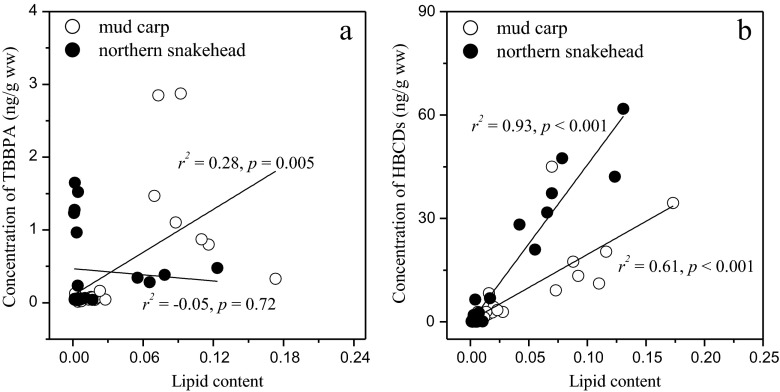
The relationship between lipid content and TBBPA (**a**), ΣHBCD (**b**) concentrations in tissues of mud carp and of northern snakehead. *ww* wet weight

A significant correlation between TBBPA level and lipid content of tissues was found for mud carp (*r*^2^ = 0.28, *p* = 0.005). However, this relationship was not found for northern snakehead (*r*^2^ = −0.05, *p* = 0.72) (Fig. 2a). This result suggested that the tissue distribution of TBBPA is not a simple passive diffusion course to lipid component which is the main tissue distribution derived for lipophobic chemicals such as polybrominated diphenyl ethers (PBDEs) (Guo et al. 2008). The tissue distributions of TBBPA in the two fish species were also different from those in fish species collected in a previous study where the tissue distribution were in the sequential order of kidney > liver > muscle > gill > spawn (Yang et al. 2012). This result suggested that the tissue distribution of TBBPA is species-specific. The significant high concentration in the scale in northern snakehead indicated that the accumulation of TBBPA in fish scale may be different from other internal tissues of fish, which could be affected more by external environments than the internal exposure.

### HBCD levels in fish tissues

The mean concentration of HBCDs in mud carp was 21.5 ng/g (ww, whole body), and that in northern snakehead was 42.8 ng/g (ww, whole body), which was 2–3 orders of magnitude greater than the mean concentration of TBBPA in mud carp (0.84 ng/g, ww) and in northern snakehead (0.38 ng/g, ww) when whole body (not accounting for tissues) was considered. The result of higher HBCD concentrations than TBBPA concentrations is similar to the results of the previous studies (Johnson-Restrepo et al. 2008; Morris et al. 2004), which can be due to the different bioaccumulation potential of the two BFRs. As previously mentioned, TBBPA is more polar and a more reactive compound compared to HBCDs, which might result in a low degree of bioaccumulation. Moreover, the higher burden of HBCDs in northern snakehead (42.8 ng/g, ww) than in mud carp (21.5 ng/g, ww) could be attributed to a higher trophic level occupied by northern snakehead than by mud carp because biomagnification of HBCDs through the aquatic food web was reported in various studies (Morris et al. 2004).

Further more, the lipid-normalized concentration of HBCDs in mud carp was 373.5 ng/g (lw, whole body), and that in northern snakehead was 385.9 ng/g (lw, whole body), which was much greater than the mean concentration of HBCDs in water (0.06 ng/L) collected from the same pond in our previous study (Wu et al. 2010). Hence, the bioconcentration factors (BCFs) would be up to 6225 and 6431 for mud carp and northern snakehead, respectively, indicating the high accumulation of HBCDs in both fish species.

Comprehensive reviews of environmental levels of HBCDs were published, e.g., by Law et al. (Law et al. 2014) and Covaci et al. (Covaci et al. 2006). The muscle is the most characterized tissue in previous studies, so a comparison of ΣHBCD concentrations in fish muscle described in lipid weight or wet weight was conducted. The ΣHBCD concentrations in fish muscle were ranged from 0.03 to 0.55 ng/g ww (43 to 120 ng/g, lw) in this study, which were much higher than those (ranged from not detected to 0.19 ng/g ww) in 12 consumer fish species from South China (Meng et al. 2012) and those (ranged from 0.57 to 10 ng/g, lw) in two species of marine fish from nine Chinese coastal cities (Xia et al. 2011). The average ΣHBCD in the northern snakehead (75 ng/g lipid) was much higher than that of freshwater fish (average 3 ng/g lw in whitefish to 15 ng/g lw in goldeye) from Lake Winnipeg in Canada (Tomy et al. 2004). However, the ΣHBCD levels in this study were lower than those (2100 to 6800 ng/g lw) in aquatic food web samples from the Western Scheldt, the Netherlands (Morris et al. 2004), those (1128 ng/g for eel muscle and 893 ng/g for trout muscle, lw) in edible fish from the riverside Skerne and Tees, UK (Allchin and Morris 2003), and those (200 to 22,000 ng/g lw) in fish samples from Etnefjorden in Norway (Köppen et al. 2010). In summary, the ΣHBCD muscle levels in the present study were at the high end of the concentration range observed in China, but it was at the low end of the concentrations observed globally.

As displayed in Fig. 1b, the ΣHBCD levels also show significant differences among tissues. The highest ΣHBCD levels occurred in chyme (96.9 ng/g, ww) of mud carp, which could be due to the residual HBCDs in the diet of mud carp. Except for chyme, fat exhibits the highest HBCD levels in both mud carp (80.9 ng/g ww) and in northern snakehead (240.2 ng/g, ww). Generally, the tissue distributions of HBCDs were similar to those of PBDEs in our previous studies (Zeng et al. 2013), in which the concentration sequential of PBDEs was fat > liver > gill > muscle. Significant correlations were found between the lipid content and the HBCD levels in mud carp (*r*^2^ = 0.78, *p* < 0.001) and in northern snakehead (*r*^2^ = 0.93, *p* < 0.001) (Fig. 2b). The result indicated that the bioaccumulation of HBCDs was a passive (lipid content-related) diffusion process.

### HBCD diastereoisomer pattern

**Fig. 3:**
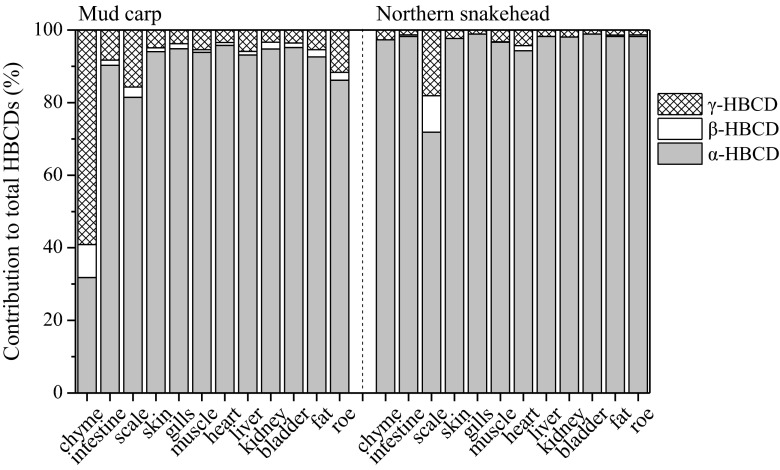
α-, β-, and γ-HBCD stereoisomer composition (%) in tissues of mud carp and of northern snakehead

α-HBCD was the predominant diastereoisomer of HBCDs in fish tissues (81.5–95.8 % in mud carp and 71.8–98.9 % in northern snakehead) except for chyme (31.8 %) of mud carp (Fig. 3), which was consistent with those in the previous studies (Morris et al. 2004; Xian et al. 2008). The preferential accumulation of α-HBCD in organisms was possibility due to a higher water solubility of α-HBCD (48.8 μg/L) than β-HBCD (14.7 μg/L) and γ-HBCD (2.1 μg/L) (Hunziker et al. 2004), the isomerization of γ-HBCD to α-HBCD (Luo et al. 2013), and a rapid elimination of β-HBCD and γ-HBCD than α-HBCD from the organisms (Law et al. 2005).

Among the tissues of the two fish species, the contribution of γ-HBCD in the scales of mud carp (15.7 %) and northern snakehead (18.1 %) was slightly higher than that of other internal tissues (3.4–11.7 % for mud carp and 1.1–4.3 % for northern snakehead) (Fig. 3), which could be a link to the partition processes of HBCDs between the water and scale. In contrast to other tissues of the two fish species, γ-HBCD was the most prominent stereoisomer (59.1 %) in the chyme of mud carp (Fig. 3). This was expected given that the existence of undigested food and the particle from the bottom of the pond in chyme, as mud carp is a filter-feeding species, feeding on organic detritus or decomposed organic matter.

### Enantiomer fractions of HBCD diastereoisomer

**Fig. 4:**
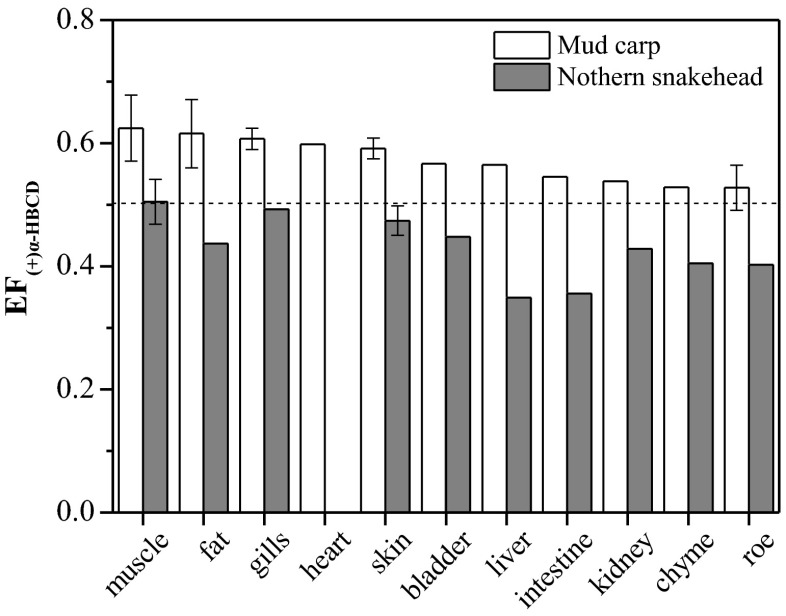
Mean enantiomeric fractions (EFs) for α-HBCD and standard deviations for different tissues in mud carp and northern snakehead

The EFs of α-HBCD were shown in Fig. 4. For β- and γ-HBCD, it was not possible to calculate EF values due to their low concentrations in most of the fish tissues. The EF values were between 0.53 and 0.62 in mud carp (Fig. 4), which were higher than those obtained from the standard solution (0.50), indicating an enrichment of (+)-α-HBCD enantiomer in mud carp. With regard to the northern snakehead, EF vaules of α-HBCD in tissues were between 0.35 and 0.50. Except for muscle, all EF values were less than 0.50, suggesting an enrichment of the (−)-α-HBCD enantiomer in northern snakehead. The EF values of muscle in the northern snakehead was 0.5, seeming to imply that no enantioselective biotransformation occurred. However, considering that mud carp was a food item of northern snakehead, the decreased EF values in northern snakehead compared with those in mud carp suggested an enantioselective accumulation occurred although the EF was 0.5. A similar result was also found in chiral polychlorinated biphenyl congeners (Tang et al. 2014). This result indicated that EF values itself cannot provide whether enantioselective processes occurred or not for a given species, a definitive conclusion can only be drawn when we obtain the original EF values for a chiral chemical.

Our results indicated that the enantiomer accumulation of HBCDs was species-dependent. This was consistent with previous reports. For example, EF_α_ values ranged between 0.23 and 0.24 for Swedish herring (Janák et al. 2008), 0.42 and 0.43 for sole liver and muscle (Janák et al. 2005), while EF_α_ values >0.5 for eel muscle (0.54), bib (0.58), and whiting liver (0.70) (Janák et al. 2005). In other studies, a significant deviation from the racemic mixture (EF = 0.5) has also been observed; EF_α_ values ranged between 0.403 and 0.476 for fish from Etnefjorden, Norway (Köppen et al. 2010), and EF_α_ >0.5 for a marine food web (Tomy et al. 2008).

## Conclusions

In summary, the tissue distribution of TBBPA and HBCDs in two fish species from an e-waste area was investigated. Passive diffusion course to lipid component is the main tissue distribution derived for HBCDs but not for TBBPA, which is likely due to the different chemical properties between chemicals. The scale exhibited a high TBBPA concentration in northern snakehead and exhibited different HBCD diastereoisomer profiles from those in other tissues which can be attributed to effects of the external environment such as water. The EF values of α-HBCD in all tissues of mud carp were lower than 0.50 whereas these were higher than 0.50 for most of the tissues in northern snakehead, indicating that the two fish species enantioselectively accumulate and/or degrade different enantiomers of α-HBCD. This result hinted that one cannot draw a definitive conclusion on whether an enantioselective degradation occurred only by an EF value itself for a given species in field study. When prey and predator metabolize different enantiomers of chiral chemicals, the EF should be interpreted carefully to avoid false conclusion.
